# 730 Does Post-hospitalization Outreach Create a Sustainable Burn Survivor Community?

**DOI:** 10.1093/jbcr/irae036.273

**Published:** 2024-04-17

**Authors:** Rana M Stephan, Vina Vargas, Clifford C Sheckter

**Affiliations:** Santa Clara Valley Medical Center, San Jose, CA; Stanford/Santa Clara Valley Medical Center, San Jose, CA; Santa Clara Valley Medical Center, San Jose, CA; Stanford/Santa Clara Valley Medical Center, San Jose, CA; Santa Clara Valley Medical Center, San Jose, CA; Stanford/Santa Clara Valley Medical Center, San Jose, CA

## Abstract

**Introduction:**

Burn survivors face many challenges after injury. Physical challenges include persistent wounds, scar contractures, thermal body dysregulation, and loss of limb. The psychological challenges can be equally challenging. Confronting isolation, depression, anxiety, post-traumatic stress, guilt, and change in body image are some of the many obstacles burn survivors face. However, a burn survivor’s participation in a burn community has shown to help alleviate the stress of reintegration by improving social interaction, maintaining friendships, improving body image, and giving tools to communicate. We hypothesize that post-hospital outreach develops a sustainable community for burn survivors.

**Methods:**

Burn survivor outreach begins during hospitalization. Burn center staff are versed on the spectrum of outreach services, and patients are invited to partake in events, as appropriate. Support group is the primary introduction to outreach services, as patients and survivors are invited to a monthly group. Additionally, post-hospital outreach consists of regular community events, recreational programs, one-on-one peer support, virtual support group and volunteer opportunities. Participation in burn outreach is voluntary at the time of hospitalization and at discharge, with patients agreeing to receive monthly burn newsletter with information about community outreach events, including support group. All voluntary participants were reviewed from 2018 – 2023 from an ABA verified burn center. Survivor community sustainability was measured by the number of survivors coming to support group.

**Results:**

Support groups were held monthly from 2018-2023, totally 69 sessions involving 61 burn staff. During the COVID-19 pandemic, support group was adapted to online version for 20 months. Post-pandemic, support group has been conducted hybrid (virtual and in-person). 372 burn survivors agreed to receive information about community outreach and support group of which 49% (182) participated. Of the 182 that participated, 111 were female and 71 were male. 11 brought family to support group. 113 participants only attended one session, while 90 participants attended two or more. Attendance ranged from 2 to 50, with a median of 25 in attendance. The median number of sessions attended per survivor was 4.0 for all-comers and 9.2 for those who attended 2 or more sessions.

**Conclusions:**

Burn survivor support group demonstrated consistent participation validating its purpose. Through building a community, psychological healing continues post-hospitalization. Furthermore, the sense of burn community is deepened by not only by continued participation in group but also by survivors extending into other outreach events.

**Applicability of Research to Practice:**

Regular support group creates a sustainable burn survivor community for those interested in participation. The hybrid format is conducive to many lifestyles and can be adopted by burn centers across the US.

